# Neuregulin 4 Is a Novel Marker of Beige Adipocyte Precursor Cells in Human Adipose Tissue

**DOI:** 10.3389/fphys.2019.00039

**Published:** 2019-01-31

**Authors:** Ferran Comas, Cristina Martínez, Mònica Sabater, Francisco Ortega, Jessica Latorre, Francisco Díaz-Sáez, Julian Aragonés, Marta Camps, Anna Gumà, Wifredo Ricart, José Manuel Fernández-Real, José María Moreno-Navarrete

**Affiliations:** ^1^Department of Diabetes, Endocrinology and Nutrition, Institut d’Investigació Biomèdica de Girona, Girona, Spain; ^2^CIBEROBN (CB06/03/010), Instituto de Salud Carlos III, Madrid, Spain; ^3^Department of Biochemistry and Molecular Biomedicine, Faculty of Biology, Institute of Biomedicine of the University of Barcelona, Barcelona, Spain; ^4^CIBER de Diabetes y Enfermedades Metabólicas Asociadas, Instituto de Salud Carlos III, Madrid, Spain; ^5^Research Unit, Hospital of Santa Cristina, Research Institute Princesa, Autonomous University of Madrid, Madrid, Spain; ^6^CIBER de Enfermedades Cardiovasculares, Carlos III Health Institute, Madrid, Spain; ^7^Department of Medicine, University of Girona, Girona, Spain

**Keywords:** obesity, neuregulin 4, browning, adipose tissue, insulin sensitivity

## Abstract

**Background:** Nrg4 expression has been linked to brown adipose tissue activity and browning of white adipocytes in mice. Here, we aimed to investigate whether these observations could be translated to humans by investigating *NRG4* mRNA and markers of brown/beige adipocytes in human visceral (VAT) and subcutaneous adipose tissue (SAT). We also studied the possible association of *NRG4* with insulin action.

**Methods:** SAT and VAT *NRG4* and markers of brown/beige (*UCP1, UCP3, and TMEM26*)-related gene expression were analyzed in two independent cohorts (*n* = 331 and *n* = 59). Insulin resistance/sensitivity was measured using HOMA_IR_ and glucose infusion rate during euglycemic hyperinsulinemic clamp.

**Results:** In both cohort 1 and cohort 2, *NRG4* and thermogenic/beige-related gene expression were significantly increased in VAT compared to SAT. Adipogenic-related genes followed an opposite pattern. In cohort 1, VAT *NRG4* gene expression was positively correlated with BMI and expression of *UCP1, UCP3, TMEM26*, and negatively with adipogenic (*FASN, PPARG*, and *SLC2A4*)- and inflammatory (*IL6 and IL8*)-related genes. In SAT, *NRG4* gene expression was negatively correlated with HOMA_IR_ and positively with *UCP1* and *TMEM26* gene expression. Multiple linear regression analysis revealed that expression of *TMEM26* gene was the best predictor of *NRG4* gene expression in both VAT and SAT. Specifically, *NRG4* and *TMEM26* gene expression was significantly increased in VAT, but not in SAT stromal vascular fraction cells (*p* < 0.001). In cohort 2, the significant association between *NRG4* and *TMEM26* gene expression in both VAT and SAT was confirmed, and SAT *NRG4* gene expression also was positively correlated with insulin action and the expression of *UCP1*.

**Conclusion:** Current findings suggest *NRG4* gene expression as a novel marker of beige adipocytes in human adipose tissue.

## Introduction

The modulation of brown adipose tissue activity and browning of white adipose tissue has been proposed as a promising therapeutic strategy in the treatment of obesity-associated metabolic disturbances (Wu et al., 2012; Bartelt and Heeren, 2014; Hepler et al., 2017; Rabhi et al., 2018; Zhang S. et al., 2018), with the intention of improving insulin sensitivity (Hepler et al., 2017; Rabhi et al., 2018) and hepatic steatosis (Huang et al., 2017), among others.

Several studies pointed to neuregulins as an important family of ligands that regulate diverse aspects of glucose and lipid metabolism and energy balance. In skeletal muscle cells, recombinant neuregulin administration stimulated glucose uptake in muscle cells (Suárez et al., 2001) in an alternative insulin-independent mechanism, activating PI3K, PDK1, and PKCzeta pathways (Cantó et al., 2004), and promoted glucose and palmitate oxidation, enhancing mitochondrial oxidative capacity (Cantó et al., 2007). In liver, neuregulin 1 (Nrg1) and neuregulin 4 (Nrg4) reduced gluconeogenesis and lipogenesis and increased fatty acid oxidation, improving systemic insulin sensitivity and glucose tolerance (Wang et al., 2014; Ennequin et al., 2015; Ma et al., 2016; Chen et al., 2017; Zhang P. et al., 2018). In fact, the Nrg4/ErbB4 signaling pathway protects hepatocytes from stress-induced cell death, preventing the steatosis to steatohepatitis progression (Guo et al., 2017). In human breast cancer cells, NRG1 binding to ERBB4 activates SREBP-2 and led to increased expression of LDL uptake- and cholesterol biosynthesis-related genes (Haskins et al., 2015). A recent study demonstrated that ErbB4 deletion accelerated the development of obesity, dyslipidemia, hepatic steatosis, hyperglycemia, hyperinsulinemia and insulin resistance after 24 week on a medium-fat diet (Zeng et al., 2018). Nrg4, a specific ligand for ErbB4 involved in neurite growth, administration in 3T3-L1 adipocytes inhibited lipogenesis and induced browning and glucose uptake, but did not exert any effects on adipogenesis and lipolysis (Zeng et al., 2018). In fact, Nrg4 has been proposed as a marker of brown adipose tissue (BAT) activity in mice, being highly expressed in cold-induced BAT activity and white adipose tissue (WAT) browning (Rosell et al., 2014; Wang et al., 2014). NRG4 was expressed in fully differentiated brown adipocytes, but not in preadipocytes, and increased during brown adipocyte differentiation (Rosell et al., 2014; Wang et al., 2014). *In vitro* experiments showed that brown adipocytes-derived NRG4 might promote the growth of neurites in adipose tissue, increasing sympathetic innervation, enhancing BAT activity and browning of WAT (Rosell et al., 2014). However, Wang et al. (2014) reported that despite the abundant expression of Nrg4 in BAT, it seems dispensable for cold-induced hypothermia response, being Ucp1 and Dio2 induced to similar extent by cold exposure in WT and NRG4KO mice. These findings indicated that Nrg4 did not directly participate in BAT thermogenesis.

Diet-induced obesity led to a significant decreased Nrg4 gene expression in WAT but not BAT (Wang et al., 2014; Ma et al., 2016; Chen et al., 2017) in mice. A recent study also showed that diet-induced non-alcoholic steatohepatitis (NASH) resulted in a significant reduced Nrg4 in both BAT and WAT (Guo et al., 2017). These studies suggested that adipose tissue-derived Nrg4 could exert positive effects on obesity associated metabolic disturbances (Wang et al., 2014; Ma et al., 2016; Chen et al., 2017; Guo et al., 2017), improving glucose tolerance and insulin sensitivity and attenuating adipose tissue and liver inflammation (Wang et al., 2014; Ma et al., 2016; Chen et al., 2017; Guo et al., 2017).

In humans, only one study investigates *NRG4* mRNA levels in adipose tissue in association with body fat mass, liver lipid content and glucose tolerance (Wang et al., 2014), but no previous studies investigated the relationship between NRG4 and markers of adipose tissue browning in human adipose tissue. Since previous mice studies demonstrated that NRG4 was a marker of BAT activity and browning of WAT (Rosell et al., 2014; Wang et al., 2014; Ma et al., 2016; Chen et al., 2017), in the present study we aimed to investigate the potential relationship between human adipose tissue NRG4 and markers of brown/beige adipocytes. Furthermore, the impact of adipose tissue NRG4 on human obesity and insulin sensitivity was also evaluated.

## Materials and Methods

### Human Adipose Tissue Samples

In cohort 1, a group of 331 [155 visceral (VAT) and 176 subcutaneous (SAT) adipose tissues] (Cohort 1) from participants with normal body weight and different degrees of obesity, with body mass index (BMI) within 20 and 68 kg/m^2^, were analyzed. In a second cohort of morbidly obese (BMI > 35 kg/m^2^) subjects with different degrees of insulin action [measured using hyperinsulinemic-euglycemic clamp (Moreno-Navarrete et al., 2013)], VAT (*n* = 34) and SAT (*n* = 25) samples (Cohort 2) were studied. Altogether these subjects were recruited at the Endocrinology Service of the Hospital of Girona “Dr Josep Trueta.” All subjects were of Caucasian origin and reported that their body weight had been stable for at least 3 months before the study. Subjects were studied in the post-absorptive state. BMI was calculated as weight (in kg) divided by height (in m) squared. They had no systemic disease other than obesity and all were free of any infections in the previous month before the study. Liver diseases (specifically tumoral disease and HCV infection) and thyroid dysfunction were specifically excluded by biochemical work-up. Samples and data from patients included in this study were partially provided by the *FATBANK* platform promoted by the *CIBEROBN* and coordinated by the *IDIBGI Biobank* (Biobank *IDIBGI*, B.0000872), integrated in the Spanish National Biobanks Network and they were processed following standard operating procedures with the appropriate approval of the Ethics, External Scientific and FATBANK Internal Scientific Committees.

#### Ethics Statement

This study was carried out in accordance with the recommendations of the ethical committee of the Hospital of Girona “Dr Josep Trueta.” The protocol was approved by the ethical committee of the Hospital of Girona “Dr Josep Trueta.” All subjects gave written informed consent in accordance with the Declaration of Helsinki, after the purpose of the study was explained to them.

AT samples were obtained from SAT and VAT depots during elective surgical procedures (cholecystectomy, surgery of abdominal hernia and gastric bypass surgery). Adipose tissue samples were washed, fragmented and immediately flash-frozen in liquid nitrogen before being stored at -80°C.

The isolation of adipocyte and stromal vascular fraction cells (SVF) was performed from 17 SAT and 20 VAT non-frozen adipose tissue samples. These samples were washed three to four times with phosphate-buffered saline (PBS) and suspended in an equal volume of PBS supplemented with 1% penicillin-streptomycin and 0.1% collagenase type I prewarmed to 37°C. The tissue was placed in a shaking water bath at 37°C with continuous agitation for 60 min and centrifuged for 5 min at 400 *g* at room temperature. The supernatant, containing mature adipocytes, was recollected. The pellet was identified as the SVF. Isolated mature adipocytes and SVF stored at -80°C for gene expression analysis.

### Analytical Methods

Serum glucose concentrations were measured in duplicate by the glucose oxidase method using a Beckman glucose analyser II (Beckman Instruments, Brea, CA, United States). Intraassay and interassay coefficients of variation were less than 4% for all these tests. HDL cholesterol was quantified by a homogeneous enzymatic colorimetric assay through the cholesterol esterase/cholesterol oxidase/peroxidase reaction (Cobas HDLC3). Total serum triglycerides were measured by an enzymatic, colorimetric method with glycerol phosphate oxidase and peroxidase (Cobas TRIGL). We used a Roche Hitachi Cobas c 711 instrument to perform the determinations.

### RNA Expression

RNA purification was performed using the RNeasy Lipid Tissue Mini Kit (QIAGEN, Izasa SA, Barcelona, Spain) and the integrity was checked by the Agilent Bioanalyzer (Agilent Technologies, Palo Alto, CA, United States). Gene expression was assessed by real time PCR using a LightCycler^®^ 480 Real-Time PCR System (Roche Diagnostics SL, Barcelona, Spain), using TaqMan^®^ and SYBR green technology suitable for relative genetic expression quantification. The RT-PCR reaction was performed in a final volume of 12 μl. The cycle program consisted of an initial denaturing of 10 min at 95°C then 40 cycles of 15 s denaturizing phase at 95°C and 1 min annealing and extension phase at 60°C. A threshold cycle (Ct value) was obtained for each amplification curve and a ΔCt value was first calculated by subtracting the Ct value for human cyclophilin A (*PPIA*) RNA from the Ct value for each sample. Fold changes compared with the endogenous control were then determined by calculating 2^-ΔCt^, so that gene expression results are expressed as expression ratio relative to *PPIA* gene expression according to the manufacturer’s guidelines. PPIA Ct values in both SAT and VAT were comparable (23.48 ± 0.81 in SAT vs. 23.49 ± 1.28 in VAT, *p* = 0.9, *n* = 152). Primer/probe sets used were: neuregulin 4 (*NRG4*, Hs00163592_m1), fatty acid synthase (*FASN*, Hs00188012_m1), peroxisome proliferator-activated receptor gamma (*PPARG*, Hs00234592_m1), solute carrier family 2 (facilitated glucose transporter), member 4 (*SLC2A4* or *GLUT4*, Hs00168966_m1), perilipin 1 (*PLIN1*, Hs00160173_m1), PPARG coactivator 1 alpha (*PPARGC1A*, Hs00173304_m1), uncoupling protein 1 (*UCP1*, Hs01084772_m1), uncoupling protein 3 (*UCP3*, Hs01106052_m1), transmembrane protein 26 (*TMEM26*, Hs00415619_m1), interleukin 6 (*IL6*, Hs00174131_m1), C-X-C motif chemokine ligand 8 (*CXCL8* or also named IL8, Hs00174103_m1), and peptidylprolyl isomerase A (cyclophilin A) (4333763, *PPIA* as endogenous control).

### Statistical Analyses

Statistical analyses were performed using the SPSS 12.0 software. Unless otherwise stated, descriptive results of continuous variables are expressed as mean and SD for Gaussian variables or median and interquartile range for non-Gaussian variables. Parameters that did not fulfill normal distribution criteria were log transformed to improve symmetry for subsequent analyses. The relation between variables was analyzed by simple correlation (using Spearman’s and Pearson’s tests) and multiple linear regression analyses. ANOVA and unpaired Student’s *t*-tests were used to compare clinical variables and gene expression relative to obesity and type 2 diabetes (T2D).

## Results

Representative Ct values of analyzed genes were shown in Table 1.

**Table 1:** Representative Ct values of analyzed genes.

	Mean ± SD
*PPIA*	23.22 ± 0.25
*FASN*	25.91 ± 1.83
*PPARG*	29.49 ± 0.85
*SLC2A4*	27.71 ± 0.53
*PLIN1*	22.78 ± 0.49
*PPARGC1A*	30.86 ± 0.71
*UCP1*	36.82 ± 0.91
*UCP3*	34.11 ± 0.65
*TMEM26*	34.16 ± 0.95
*IL6*	31.81 ± 2.28
*IL8*	30.89 ± 1.92
*NRG4*	35.57 ± 0.99


### Cohort 1

**FIGURE 1:**
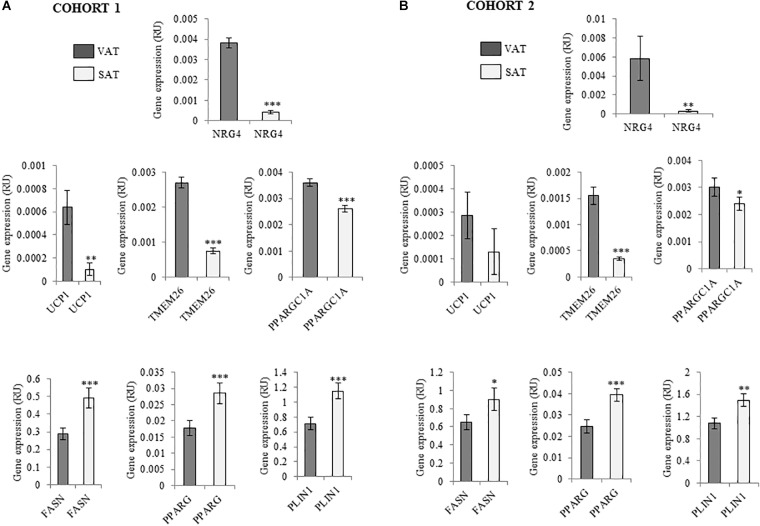
**(A,B)** Comparison of *NRG4, UCP1, TMEM26, PPARGC1A, FASN, PPARG*, and *PLIN1* gene expression in paired VAT and SAT in cohort 1 (*n* = 132) **(A)** and cohort 2 (*n* = 20) **(B)**. ^∗^*p* < 0.05, ^∗∗^*p* < 0.01, and ^∗∗∗^*p* < 0.001 compared to gene expression in VAT.

**FIGURE 2:**
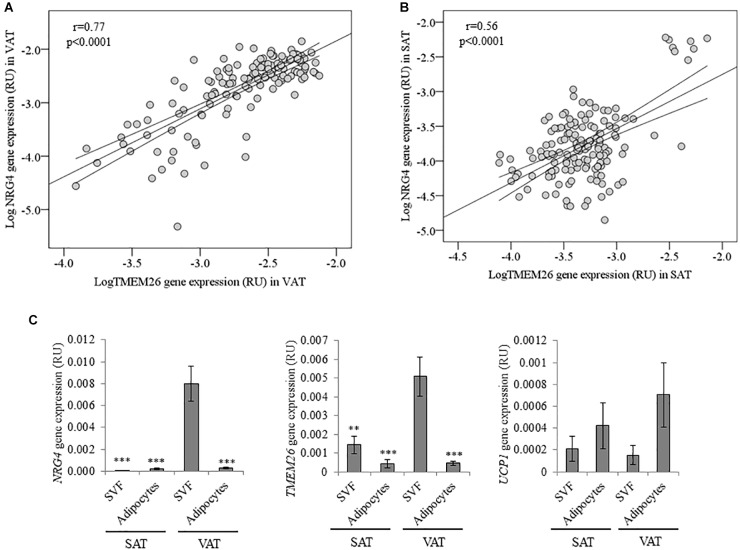
**(A,B)** Bivariate correlation between *NRG4* and *TMEM26* gene expression in VAT **(A)** and SAT **(B)**. **(C)** *NRG4, TMEM26*, and *UCP1* gene expression in adipose tissue fractions (SVF and adipocytes) in both SAT (*n* = 17) and VAT (*n* = 20). ^∗∗^*p* < 0.01 and ^∗∗∗^*p* < 0.001 compared to gene expression in VAT SVF cells.

Anthropometric and clinical data from cohort 1 were detailed in Table 2. Similar to thermogenic/beige-related gene expression, *NRG4* was significantly increased in VAT compared to SAT, whereas adipogenesis-related genes followed an opposite gene expression pattern (Figure 1A). In cohort 1, VAT *NRG4* gene expression was significantly increased in participants with obesity (Table 2), but no significant differences were found between non-diabetic obese and obese participants with T2D (Table 2). No significant differences were observed on SAT *NRG4* gene expression according to obesity or T2D. In VAT, *NRG4* gene expression was positively correlated with BMI, and negatively correlated with adipogenic-related genes (*FASN, PPARG*, and *SLC2A4*) (Table 3). Interestingly, *NRG4* gene expression was significantly positively associated with expression of brown/beige adipocyte activity-related (*UCP1, UCP3, and TMEM26*) and negatively with inflammatory-related (*IL6 and IL8*) genes (Table 3 and Figure 2A). In SAT, *NRG4* gene expression was negatively correlated with HOMA_IR_ and positively with *UCP1* and *TMEM26* gene expression (Table 3 and Figure 2B).

**Table 2:** Anthropometric and clinical characteristics according to obesity and T2D in cohort 1.

	Non-obese	Obese	Obese + T2D	*p*
N	54	88	34	
Age (years)	47.4 ± 10.1	45.6 ± 10.5	47.2 ± 9.5	0.5
BMI (kg/m^2^)	25.4 ± 3.8	43.9 ± 7.4^∗^	44.7 ± 4.1^∗^	**<0.0001**
Fasting glucose (mg/dl)^a^	86 (80–94)	93 (84–100.5)	126 (93.5–169.5)^∗#^	**<0.0001**
HOMA_IR_ (*n* = 56)^a^	1.18 (0.79–1.76)	2.06 (1.44–3.39)	5.59 (3.93–7.05)^∗#^	**0.001**
Total-cholesterol (mg/dl)^a^	199 (174–219)	193 (167.5–218.7)	182 (166–214)	0.5
HDL-cholesterol (mg/dl)^a^	64.5 (50.7–77.5)	55 (45.5–62.6)	50.1 (42–62)^∗^	**0.04**
LDL-cholesterol (mg/dl)^a^	114.5 (88.7–135.5)	116.8 (97.5–134.7)	101.5 (89.5–137.7)	0.5
Fasting triglycerides (mg/dl)^a^	79.5 (57.7–101.2)	98 (75–132)	136 (89.5–164.5)^∗#^	**<0.0001**
VAT *NRG4* (RU) ×10^-3a^	1.26 (0.217–4.11)	3.59 (2.24–5.56)^∗^	4.58 (2.54-6.52)^∗^	**0.001**
SAT *NRG4* (RU) ×10^-3a^	0.141 (0.061–0.239)	0.168 (0.095–0.381)	0.083 (0.057–0.155)	0.2


*VAT, visceral adipose tissue; SAT, subcutaneous adipose tissue; T2D, type 2 diabetes; HOMA_IR_, homeostasis model assessment – insulin resistance index; RU, relative gene expression units.*

*^a^Median and interquartile range.*

*^∗^p < 0.05 compared to non-obese participants after performing Bonferroni post hoc test.*

*^#^p < 0.05 compared to obese participants after performing Bonferroni post hoc test.*

*Bold values mean that p-value reached statistical significance.*

**Table 3:** Correlation between *NRG4* gene expression and anthropometric and clinical characteristics and selected gene expression in SAT (*n* = 176) and VAT (*n* = 155) from cohort 1.

	VAT		SAT	
		
	*r*	*P*	*r*	*p*
Age (years)	-0.02	0.7	0.11	0.2
BMI (kg/m^2^)	0.28	**<0.0001**	0.06	0.5
Fasting glucose (mg/dl)	0.17	**0.03**	0.01	0.8
HOMA_IR_ (*n* = 56)	0.17	0.3	-0.32	**0.02**
Total cholesterol (mg/dl)	-0.10	0.2	0.11	0.2
HDL cholesterol (mg/dl)	-0.01	0.9	0.15	0.1
LDL cholesterol (mg/dl)	-0.09	0.3	0.08	0.4
Fasting triglycerides (mg/dl)	0.05	0.5	-0.09	0.3
*FASN* (RU)	-0.37	**<0.0001**	-0.05	0.6
*PPARG* (RU)	-0.39	**<0.0001**	0.05	0.6
*SLC2A4* (RU)	-0.33	**0.002**	0.12	0.2
*PLIN1* (RU)	-0.15	0.1	-0.10	0.4
*PPARGC1A* (RU)	0.06	0.5	0.18	0.05
*UCP1* (RU)	0.30	**0.005**	0.30	**0.001**
*UCP3* (RU)	0.29	**0.005**	0.13	0.1
*TMEM26* (RU)	0.77	**<0.0001**	0.56	**<0.0001**
*IL6* (RU)	-0.45	**<0.0001**	0.06	0.5
*IL8* (RU)	-0.36	**0.001**	-0.05	0.6


*Bold values mean that p-value reached statistical significance.*

In multiple linear regression analysis, *TMEM26* (β = 0.58, *p* < 0.0001; model adjusted *R*^2^ = 0.37, *p* < 0.0001), *UCP3* (β = 0.24, *p* = 0.03; model adjusted *R*^2^ = 0.13, *p* = 0.001), *IL6* (β = -0.32, *p* = 0.01; model adjusted *R*^2^ = 0.16, *p* = 0.001), *IL8* (β = -0.36, *p* = 0.008; model adjusted *R*^2^ = 0.17, *p* < 0.0001), *FASN* (β = -0.42, *p* = 0.001, model adjusted *R*^2^ = 0.12, *p* = 0.001) and *PPARG* (β = -0.38, *p* = 0.005; model adjusted *R*^2^ = 0.11, *p* = 0.008) significantly contributed to the variance of *NRG4* gene expression in VAT after controlling for BMI. In SAT, *TMEM26* (β = 0.77, *p* < 0.0001; model adjusted *R*^2^ = 0.61, *p* < 0.0001) significantly contributed to the variance of *NRG4* gene expression after controlling for BMI. Multiple linear regression analysis revealed that expression of *TMEM26* gene was the best predictor of *NRG4* gene expression in both VAT and SAT.

In addition, correlations between UCP1, a specific marker of brown adipocytes, and clinical and metabolic parameters were also explored. No significant correlation between SAT or VAT *UCP1* gene expression and BMI, fasting glucose, HOMA_IR_, total-, LDL- and HDL-cholesterol, and fasting triglycerides were observed. VAT, but not SAT, *UCP1* was positively correlated with *SLC2A4* (*r* = 0.43, *p* < 0.0001), *PPARGC1A* (*r* = 0.36, *p* = 0.001) and *UCP3* (*r* = 0.31, *p* = 0.005) gene expression.

In adipose tissue fractions, *NRG4* and *TMEM26*, but not *UCP1*, gene expression was significantly increased in visceral SVFs compared to visceral adipocytes, subcutaneous SVFs and subcutaneous adipocytes (Figure 2C).

### Cohort 2

To examine the findings replication of cohort 1 excluding the effects of obesity, an independent cohort (cohort 2) composed of morbidly subjects with different degrees of insulin action has been analyzed. Anthropometric and clinical data from cohort 2 were detailed in Table 4. Similar to cohort 1, *NRG4* and thermogenic/beige- related gene expression was increased in VAT (Figure 1B). No significant differences on SAT or VAT *NRG4* gene expression according to glucose tolerance or T2D were found. VAT *NRG4* gene expression were associated with expression of *TMEM26* gene (Table 5), and SAT NRG4 with insulin sensitivity (M) and expression of *SLC2A4, UCP1* and *TMEM26* genes (Table 5).

**Table 4:** Anthropometric and clinical characteristics according to glucose tolerance in cohort 2.

	NGT	IGT	T2D	*p*
	**11**	**10**	**13**	
Age (years)	41.6 ± 4.1	50 ± 8.5^∗^	51.5 ± 7.5^∗^	**0.004**
BMI (kg/m^2^)	46.3 ± 8.7	47.8 ± 3.2	44.7 ± 8.1	0.6
Fasting glucose (mg/dl)^a^	90 (83–98)	102.5 (96–107)	121 (100.5–132)^∗^	**0.003**
M [mg/(kg.min)]^a^	4.35 (2.21–6.28)	3.41 (1.92–5.21)	2.79 (1.68–4.31)	0.6
Total-cholesterol (mg/dl)^a^	182 (163–221)	207 (184.5–252)	179 (162–209)	0.07
HDL-cholesterol (mg/dl)^a^	49 (40–58)	45 (34.5–55.5)	46 (40.5–49.5)	0.5
LDL-cholesterol (mg/dl)^a^	105 (95–129)	147 (110.7–176.2)	108 (84.5–133)	**0.03**
Fasting triglycerides (mg/dl)^a^	99 (68–134)	141.5 (66.7–190.7)	139 (87.5–183)	0.4
VAT *NRG4* (RU) ×10^-3a^	4.04 (1.11–6.08)	2.97 (0.141–5.92)	3.37 (2.14–6.17)	0.4
SAT *NRG4* (RU) ×10^-3a^	0.172 (0.072–0.287)	0.099 (0.081–0.129)	0.131 (0.039–0.182)	0.4


*VAT, visceral adipose tissue; SAT, subcutaneous adipose tissue; NGT, normal glucose tolerance; IGT, impaired glucose tolerance; T2D, type 2 diabetes; M, insulin sensitivity obtained from hyperinsulinemic-euglycemic clamp; RU, relative gene expression units.*

*^a^Median and interquartile range.*

*^∗^p < 0.05 compared to NGT participants after performing Bonferroni post hoc test.*

*Bold values mean that p-value reached statistical significance.*

**Table 5:** Correlation between *NRG4* gene expression and anthropometric and clinical characteristics and selected gene expression in SAT (*n* = 25) and VAT (*n* = 34) from cohort 2.

	VAT		SAT	
		
	*r*	*p*	*r*	*p*
Age (years)	-0.07	0.7	-0.17	0.4
BMI (kg/m^2^)	0.06	0.7	-0.08	0.7
Fasting glucose (mg/dl)	0.13	0.4	-0.21	0.3
M [mg/(kg.min)]	-0.03	0.9	0.43	**0.04**
Total cholesterol (mg/dl)	-0.23	0.2	-0.22	0.3
HDL cholesterol (mg/dl)	0.07	0.7	0.11	0.5
LDL cholesterol (mg/dl)	-0.07	0.7	-0.27	0.2
Fasting triglycerides (mg/dl)	-0.14	0.4	-0.14	0.5
*FASN* (RU)	0.05	0.8	0.13	0.6
*PPARG* (RU)	-0.09	0.6	0.17	0.4
*SLC2A4* (RU)	-0.09	0.6	0.49	**0.01**
*PLIN1* (RU)	0.04	0.8	-0.15	0.5
*PPARGC1A* (RU)	0.37	0.1	0.04	0.8
*UCP1* (RU)	0.29	0.1	0.43	**0.04**
*UCP3* (RU)	0.13	0.6	0.03	0.8
*TMEM26* (RU)	0.56	**0.002**	0.51	**0.01**
*IL6* (RU)	0.03	0.9	-0.10	0.6


*Bold values mean that p-value reached statistical significance.*

## Discussion

To the best of our knowledge, this is the first study showing a significant relationship between *NRG4* and *TMEM26* gene expression in human adipose tissue. Interestingly, this association was found in both VAT and SAT, and validated in a second independent cohort. TMEM26 has been described as a specific marker of brite/beige adipocytes (Wu et al., 2012; Torriani et al., 2016; Finlin et al., 2017). We also found positive associations among NRG4 and markers of thermogenic activity (characteristic of both brown and beige adipocytes) such as expression of *UCP1* and *UCP3* genes. In addition, VAT *NRG4* gene expression was negatively correlated with expression of white lipogenic/adipogenic (*FASN and PPARG*)- and inflammatory (*IL6 and IL8*)-related genes, even after controlling for BMI. Since beige adipocytes have less lipogenic capacity compared to white adipocytes (Aziz et al., 2017; Zuriaga et al., 2017), and browning/beiging of adipose tissue protected against visceral adipose tissue inflammation (Wu et al., 2017; Gonzalez-Hurtado et al., 2018), the negative association between NRG4 and white adipogenic/inflammatory genes reinforced NRG4 as a marker of beige adipocytes. In fact, these correlations were only observed in the samples with the highest correlation between NRG4 and TMEM26 (*r* = 0.77, *p* < 0.0001). However, the correlations between VAT *NRG4* and *UCP1*, *UCP3*, lipogenic/adipogenic - and inflammatory-related gene expression were not replicated in morbidly obese participants (cohort 2). Further studies in human adipose tissue should be required to validate these correlations.

Furthermore, in both cohort 1 and 2, similar to beige adipocytes-related genes (TMEM26), *NRG4* gene expression was significantly more expressed in VAT, whereas, as expected adipogenic-related genes were more expressed in SAT (Sauma et al., 2007; Moreno-Navarrete et al., 2016; Zuriaga et al., 2017). Contrary to mice, increased pattern of browning gene expression in human VAT compared to SAT has been reported (Zuriaga et al., 2017). Interestingly, *NRG4* and *TMEM26* gene expression was enriched in SVFs from VAT compared to SVFs from SAT or adipocytes from VAT or SAT. This finding points to a specific population of beige precursor cells in VAT, characterized by increased *NRG4* and *TMEM26* gene expression, and could explain the increased expression of beige/browning-related genes observed in this fat depot (current data and Zuriaga et al., 2017). Reinforcing this idea, previous studies demonstrated that *TMEM26* gene expression was also increased in SVF and decreased in the late stages of beige adipocyte differentiation, and indicated its abundance in the precursors of beige adipocytes (Lee et al., 2015; Garcia et al., 2016).

Altogether these findings indicated NRG4 as an additional marker of beige adipocytes in human adipose tissue, and suggested a possible role of this factor in the development of beige adipocytes in human fat depots. Supporting this hypothesis, Rosell et al. (2014) suggested that NRG4 might promote the growth of neurites in adipose tissue, increasing sympathetic innervation and in consequence, enhancing browning of WAT. Regarding the possible role of NRG4 on thermogenic activity, Wang et al. (2014) demonstrated in Nrg4 deficient mice that Nrg4 did not directly participate in BAT thermogenesis, but Ma et al. (2016) showed that Nrg4 overexpression enhanced BAT activity with an increase of ∼1°C body temperature, and BAT and iWAT thermogenic gene expression. These studies supported a possible role of NRG4 in beiging of human adipose tissue, but contradictory data in relation to its thermogenic activity. Further functional studies in human adipose tissue should be required to confirm the possible role of NRG4 in this process.

Another interesting finding of current study was the positive association between SAT NRG4 gene expression and insulin sensitivity. In a previous study, SAT and VAT NRG4 was significantly decreased in patients with impaired glucose tolerance (IGT) and T2D (Wang et al., 2014), but this study did not evaluate insulin sensitivity. Even tough, no significant differences were found in relation to IGT or T2D, probably due to the relatively low number of adipose tissue samples compared to the previous study (*n* = 642) (Wang et al., 2014). The current study showed a positive association between SAT *NRG4* and insulin sensitivity in both cohort 1 and cohort 2, evaluated by two different methods (HOMA_IR_ in cohort 1 and hyperinsulinemic-euglycemic clamp in cohort 2). In agreement with these findings, mice studies demonstrated that liver and adipose tissue Nrg4 overexpression improved insulin sensitivity and glucose tolerance and prevented HFD-induced hyperinsulinemia (Ma et al., 2016). In fact, two recent studies (López-Soldado et al., 2016; Zhang P. et al., 2018) demonstrated that recombinant neuregulin administration improved glucose tolerance in both control and diabetic rats by enhancing hepatic glucose utilization (López-Soldado et al., 2016) and insulin sensitivity in high fat-fed mice (Zhang P. et al., 2018).

On the other hand, contrary to previous study that demonstrated that SAT NRG4 was negatively correlated with body fat mass (Wang et al., 2014), in the current study no significant association was found between SAT *NRG4* and BMI. In cohort 1, VAT *NRG4* gene expression was increased in obese compared to non-obese participants, and positively correlated with BMI, but in cohort 2, VAT *NRG4* gene expression was not correlated with BMI. Of note, similar *NRG4* gene expression values were observed comparing obese participants from cohort 1 vs. those from cohort 2. Strikingly, the positive effects of diet-induced weight loss reducing body fat mass were not associated with expression of brown/beige-related genes (Barquissau et al., 2018). However, additional studies will be necessary to clarify the relationship between human adipose tissue *NRG4* and obesity.

A significant limitation of current study was the absence of VAT or SAT NRG4 protein analysis by scarce availability of adipose tissue lysates for protein in the same tissue samples used for RNA analysis. Similar to this, NRG4 protein analysis was not evaluated in recent relevant studies that demonstrated the importance of NRG4 in adipose tissue (Wang et al., 2014; Chen et al., 2017; Guo et al., 2017; Nugroho et al., 2018; Pellegrinelli et al., 2018). Thus, additional studies should be performed to investigate if NRG4 protein follows the same pattern of mRNA expression in human adipose tissue. Interestingly and consistent with current findings, increased NRG4 mRNA and protein release in human beige adipogenesis of mural-like mesenchymal stem cell was more recently reported (Su et al., 2018), indicating that *NRG4* gene expression were correlated with NRG4 protein levels and supporting NRG4 participation in beige adipocyte differentiation. However, it is important to note that expression of *NRG4* and brown/beige adipose tissue markers (*UCP1, UCP3*, and *TMEM26*) were extremely low, suggesting that browning of white adipose tissue in humans may have less relevance than in mice.

In conclusion, all these observations suggest *NRG4* gene expression as a novel marker of beige adipocytes in human adipose tissue.

## Author Contributions

JF-R and JM-N participated in study design and analysis of data and wrote and edited the manuscript. FC, CM, MS, FO, JL, and FD-S participated in acquisition of data. JA, MC, AG, and WR participated in interpretation of data. FC, CM, MS, FO, JL, FD-S, JA, MC, AG, and WR revised the manuscript critically for important intellectual content. All authors participated in final approval of the version to be published.

## Conflict of Interest Statement

The authors declare that the research was conducted in the absence of any commercial or financial relationships that could be construed as a potential conflict of interest.
